# The Washing Machine as a Reservoir for Transmission of Extended-Spectrum-Beta-Lactamase (CTX-M-15)-Producing Klebsiella oxytoca ST201 to Newborns

**DOI:** 10.1128/AEM.01435-19

**Published:** 2019-10-30

**Authors:** Ricarda M. Schmithausen, Esther Sib, Martin Exner, Sylvia Hack, Claudia Rösing, Patrick Ciorba, Gabriele Bierbaum, Mykhailo Savin, Sally F. Bloomfield, Martin Kaase, Anja Jacobshagen, Stefanie Gemein, Jürgen Gebel, Steffen Engelhart, Daniel Exner

**Affiliations:** a Institute for Hygiene and Public Health, University Hospital Bonn, Bonn, Germany; b Institute of Medical Microbiology, Immunology, and Parasitology, Bonn, Germany; c Department of Preventive Health Management, Institute of Animal Science, Bonn, Germany; d London School of Hygiene and Tropical Medicine, University of London, London, United Kingdom; e National Reference Laboratory for Multidrug-Resistant Gram-Negative Bacteria, Department of Medical Microbiology, Ruhr University Bochum, Bochum, Germany; f Department of General, Visceral, Thoracic, and Vascular Surgery, University Hospital Bonn, Bonn, Germany; Rutgers, The State University of New Jersey

**Keywords:** ESBL-producing bacteria, *Klebsiella oxytoca*, ST201, washing machine, laundry, colonization, newborns

## Abstract

During the period from April 2012 to May 2013, 13 newborns (1 to 4 weeks of age) and 1 child in a pediatric hospital ward in Germany were colonized with Klebsiella oxytoca producing an extended-spectrum beta-lactamase (ESBL) (CTX-M-15). A microbiological source-tracking analysis with human and environmental samples was carried out to identify the source and transmission pathways of the K. oxytoca clone. In addition, different hygienic intervention methods were evaluated. K. oxytoca isolates were detected in the detergent drawer and on the rubber door seal of a domestic washer-extractor machine that was used in the same ward to wash laundry for the newborns, as well as in two sinks. These strains were typed using pulsed-field gel electrophoresis (PFGE) and multilocus sequence typing. The environmental findings were compared with those for the human strains and the isolates detected on clothing. The results from both techniques showed that the strains were identical (sequence type 201 and PFGE type 00531, a clone specific to this hospital and not previously isolated in Germany), emphasizing the washing machine as a reservoir and fomite for the transmission of these multidrug-resistant bacteria. After the washing machine was taken out of use, no further colonizations were detected during the subsequent 4-year period.

**IMPORTANCE** Washing machines should be further investigated as possible sites for horizontal gene transfer (ESBL genes) and cross-contamination with clinically important Gram-negative strains. Particularly in the health care sector, the knowledge of possible (re-)contamination of laundry (patients’ clothes and staff uniforms) with multidrug-resistant Gram-negative bacteria could help to prevent and to control nosocomial infections. This report describes an outbreak with a single strain of a multidrug-resistant bacterium (Klebsiella oxytoca sequence type 201) in a neonatal intensive care unit that was terminated only when the washing machine was removed. In addition, the study implies that changes in washing machine design and processing are required to prevent accumulation of residual water where microbial growth can occur and contaminate clothes.

## Abstract (precis)

Washing machines should be further investigated as possible sites for horizontal gene transfer (ESBL genes) and cross-contamination with clinically important Gram-negative strains. Particularly in the health care sector, the knowledge of possible (re-)contamination of laundry (patients’ clothes and staff uniforms) with multidrug-resistant Gram-negative bacteria could help to prevent and to control nosocomial infections. This report describes an outbreak with a single strain of a multidrug-resistant bacterium (Klebsiella oxytoca sequence type 201) in a neonatal intensive care unit that was terminated only when the washing machine was removed. In addition, the study implies that changes in washing machine design and processing are required to prevent accumulation of residual water where microbial growth can occur and contaminate clothes.

## INTRODUCTION

Water or wastewater contaminated with facultative pathogenic microorganisms provides a potential reservoir for infections (1–3). The most common bacteria causing health care-associated infections or permanently colonizing reservoirs linked to contaminated hospital water in the environments of patients are Gram-negative bacteria, including *Pseudomonas* spp., *Enterobacter* spp., *Serratia* spp., *Stenotrophomonas* spp., and *Klebsiella* spp. (1, 2, 4). Such water reservoirs in health care settings include faucets, sink surfaces, bathtubs, wastewater drainage system drains, sinks, showers, and toilets (4, 5). Persisting and especially multidrug-resistant (MDR) bacteria in those reservoirs pose severe risks, especially for high-risk patients such as severely immunocompromised patients, newborns (1 to 4 weeks of age), and infants (6 to 8 weeks of age) in neonatal intensive care units (ICUs) (4, 6–10). For example, Leitner et al. (11) described an outbreak of 6 infections in patients with hematological malignancies that involved a strain of KPC-2-producing Klebsiella oxytoca, which is most commonly linked to contaminated water reservoirs. Identification of the contaminated environmental reservoir in such outbreaks can be challenging, and transmission pathways have been elucidated in only a few clinical studies (6, 12, 13).

Currently, washing machines and clothing are not assessed as potential reservoirs in outbreaks of nosocomial infections, despite evidence that a potential health risk due to contaminated laundry cannot be excluded (14–16). Recently, Rehberg et al. (17) described the presence of antibiotic-resistant bacteria (ARB) and their possible transmission via washing machines.

## RESULTS

### Case description.

Between April 2012 and May 2013, increased rates of colonization with K. oxytoca isolates were recorded in a level 1 perinatal center (PNC) and in several wards in the connected children’s hospital (90 stationary beds and 20 ICU beds) in western Germany. The colonizations had been noticed after implementation of a standard screening procedure for incoming individuals and/or patients with risk factors (newborns, children, and mothers), as a general control mechanism aimed at reducing the spread of MDR bacteria. Screening included anal swabs (newborns, children, and mothers), vaginal swabs (mothers), and in some cases additional wound swabs (newborns and children).

### Colonization with K. oxytoca in humans.

Within 14 months, 27 children were colonized (but not infected) with K. oxytoca, according to CDC definitions (18). The occurrence of K. oxytoca varied from sensitive isolates with no extended-spectrum beta-lactamase (ESBL) activity to ESBL-producing K. oxytoca isolates and the particular ESBL-producing K. oxytoca pulsed-field gel electrophoresis (PFGE) type 00531. Fourteen children tested positive for PFGE type 00531 ESBL-producing K. oxytoca.

K. oxytoca was mostly identified in rectal swab screening samples. For 9 of 24 newborns, K. oxytoca was also detected in throat swab samples. As an exception, a 4-year-old boy in the ICU who never had any direct contact with the PNC also showed rectal colonization with PFGE type 00531/sequence type 201 (ST201) ESBL-producing K. oxytoca in March 2013.

**FIG 1:**
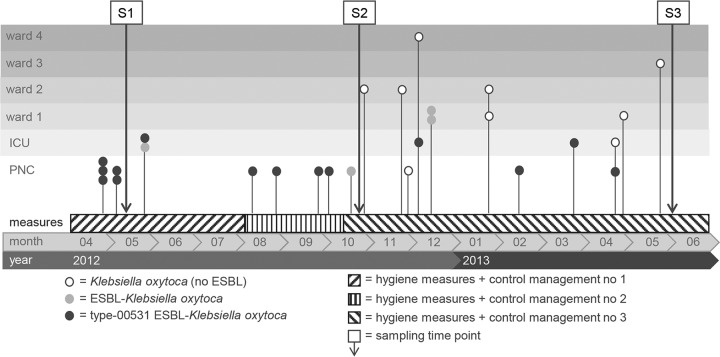
Course of the outbreak with different K. oxytoca strains over 1 year and their distributions in different wards. S1, first sampling; S2, second sampling; S3, third sampling.

All ESBL-producing K. oxytoca strains were resistant to piperacillin-tazobactam (MICs up to and higher than the measurable limit of 64/4 mg/liter), cefotaxime (MICs of ≥64 mg/liter), ceftazidime (MICs of >128 mg/liter), and ciprofloxacin (MICs of ≥2 mg/liter) and could be classified as MDR according to the guidelines reported by Magiorakos et al. (19). Figure 1 summarizes the occurrence of K. oxytoca isolates over time in the PNC, the ICU, and four different wards.

Until October 2012, only newborns in the PNC or infants in the ICU were colonized with ESBL-producing K. oxytoca, some strains of which belonged to PFGE type 00531/ST201 (Fig. 1). From the end of October 2012 to February 2013, K. oxytoca was also detected on other wards and among older pediatric patients (see above).

During an extended screening of mothers and health care workers (HCWs), a total of 695 swabs (vaginal and rectal) from 428 persons were obtained on the obstetrics ward. The screening identified 4 mothers colonized with K. oxytoca, 5 mothers colonized with ESBL-producing Escherichia coli, and 1 mother colonized with ESBL-producing Klebsiella pneumoniae. None was positive for PFGE type 00531 ESBL-producing K. oxytoca. Transmission between a mother and a newborn was not documented.

### Environmental monitoring.

All environmental samples obtained during the first and second on-site inspections tested negative for *Enterobacterales* and nonfermenting organisms. During the second on-site sampling, in October 2012, only low concentrations of Gram-positive skin and environmental bacteria were present. During the third on-site inspection, in June 2013, sampling was conducted using native samples (drinking water and wastewater) and liquid medium swabs (ESwabs). Thus, *Enterobacterales* and nonfermenting bacteria were detected. All K. oxytoca isolates were identical ESBL-producing strains and belonged to PFGE type 00531 (Table 1). In the following discussion, these K. oxytoca strains are termed environmental.

**TABLE 1:** Occurrence of Gram-negative *Enterobacterales* strains, nonfermenting organisms, and PFGE type 00531 ESBL-producing K. oxytoca isolates in environmental samples obtained during on-site inspection of risk areas

Room	Sampling point	Counts (CFU/ml or total Gram-negative bacterial count)	Microbiological differentiation	CTX-M	PFGE type 00531/ST201 K. oxytoca
Nursing care room, storage rooms	Working basin, siphon	2.1 × 10^5^	Serratia marcescens		
	Hand wash basin, siphon water	1.6 × 10^6^	K. oxytoca	Positive	Yes
			Raoultella terrigena	Negative	
			Enterobacter cloacae complex	Negative	
	Hand wash basin, drainage hole cover	Scattered/swab (no CFU/ml)	E. cloacae complex	Negative	
			K. pneumoniae	Negative	
Staff toilet	Hand wash basin, fresh water	Not evaluable/100 ml	Gram-negative nonfermenting rods		
	Hand wash basin, siphon water	1.5 × 10^4^	P. aeruginosa, S. maltophilia		
	Toilet, water	1.0	S. maltophilia		
Staff room	Kitchen sink, siphon water	3.0 × 10^5^	K. oxytoca	Positive	Yes
			P. aeruginosa	Negative	
	Water reservoir tank, espresso machine	10.0 CFU/50 ml	S. maltophilia		
Hygiene sluice, PNC	Hand wash basin, siphon water	1.3 × 10^4^	P. aeruginosa, Gram-negative nonfermenting rods		
Basement, laundry room	Tumble dryer, water tray	Not evaluable/50 ml	Gram-negative nonfermenting rods		
	Washing machine 1, residual water, rubber mantle	3.9 × 10^2^	K. oxytoca	Positive	Yes
	Washing machine 1, detergent compartment	Not evaluable/swab	K. oxytoca	Positive	Yes
			P. aeruginosa	Negative	
	Washing machine 2, detergent compartment	Not evaluable/swab	P. aeruginosa		
Laundry	Hat 1	>10^9^	K. oxytoca	Positive	Yes
	Hat 2	>10^9^	K. oxytoca	Positive	Yes
	Hat 3	>10^9^	K. oxytoca	Positive	Yes
	Hat 4	>10^9^	K. oxytoca	Positive	Yes
	Socks 1	>10^9^	K. oxytoca	Positive	Yes
	Socks 2	>10^9^	K. oxytoca	Positive	Yes

Water-associated bacteria, such as Pseudomonas aeruginosa, *Serratia* spp., *Enterobacter* spp., K. pneumoniae, and Stenotrophomonas maltophilia, were detected in the siphons of hand wash basins. Identical clones of PFGE type 00531/ST201 K. oxytoca were isolated from the siphons of two sinks in the HCW staff room and in the room used for cleaning and disinfection. The same clone was also isolated at high concentrations from samples of residual water in the rubber seal and from a swab sample (in addition to P. aeruginosa) from the detergent compartment of one of the two washing machines (Table 1) located on the ground floor of the same ward.

Following identification of the washing machine as a potential reservoir for this specific K. oxytoca clone, newborn clothing (hats and socks) that had been washed in this machine were microbiologically analyzed. PFGE type 00531/ST201 K. oxytoca of the same specific clone was isolated, with total counts of >10^9^ CFU/ml. All PFGE type 00531/ST201 ESBL-producing K. oxytoca isolates found in the residual water and environmental samples were also resistant to piperacillin-tazobactam (MICs ranging from 8/4 mg/liter to >64/4 mg/liter), cefotaxime (MICs of >2 mg/liter), ceftazidime (MICs of 64 mg/liter), and ciprofloxacin (MICs of ≥ 2 mg/liter) (Table 2).

**TABLE 2:** MICs of K. oxytoca isolates obtained during on-site inspection of risk areas

No.	Species	Sample type	MIC (mg/liter)^*a*^							
			Piperacillin	Piperacillin-tazobactam	Cefotaxime	Ceftazidime	Imipenem	Meropenem	Amikacin	Ciprofloxacin
1	K. oxytoca	Washing machine	R (>16)	I (16/4)	R (>2)	R (32)	S (≤1)	S (≤0.125)	S (≤4)	R (2)
2	K. oxytoca	Washing machine	R (>16)	R (>64/4)	R (>2)	R (32)	S (≤1)	S (≤0.125)	S (8)	R (2)
3	K. oxytoca	Washing machine	R (>16)	R (64/4)	R (>2)	R (32)	S (≤1)	S (≤0.125)	S (8)	R (2)
4	K. oxytoca	Siphon from wash basin in staff lobby	R (>16)	R (32/4)	R (>2)	R (32)	S (≤1)	S (≤0.125)	S (8)	R (>2)
5	K. oxytoca	Siphon from wash basin in staff lobby	R (>16)	S (8/4)	R (>2)	R (32)	S (≤1)	S (≤0.125)	S (≤4)	R (2)
6	K. oxytoca	Siphon from wash basin in staff lobby	R (>16)	I (16/4)	R (>2)	R (64)	S (≤1)	S (≤0.125)	S (≤4)	R (2)
7	K. oxytoca	Human	R (>16)	R (>64/4)	R (>2)	R (>128)	S (≤1)	S (≤0.125)	I (16)	R (>2)
8	K. oxytoca	Human	R (>16)	R (>64/4)	R (>2)	R (>128)	S (≤1)	S (≤0.125)	S (8)	R (>2)
9	K. oxytoca	Human	R (>16)	R (>64/4)	R (>2)	R (>128)	S (≤1)	S (≤0.125)	S (8)	R (>2)
10	K. oxytoca	Human	R (>16)	R (>64/4)	R (>2)	R (>128)	S (≤1)	S (≤0.125)	S (≤4)	R (>2)
11	K. oxytoca	Human	R (>16)	R (>64/4)	R (>2)	R (>128)	S (≤1)	S (≤0.125)	S (8)	R (>2)
12	K. oxytoca	Human	R (>16)	R (>64/4)	R (>2)	R (>128)	S (≤1)	S (≤0.125)	S (≤4)	R (>2)
13	K. oxytoca	Human	R (>16)	R (>64/4)	R (>2)	R (>128)	S (≤1)	S (≤0.125)	S (≤4)	R (2)
14	K. oxytoca	Human	R (>16)	R (>64/4)	R (>2)	R (>128)	S (≤1)	S (≤0.125)	S (≤4)	R (>2)
15	K. oxytoca	Human	R (>16)	R (>64/4)	R (>2)	R (>128)	S (≤1)	S (≤0.125)	S (8)	R (>2)
16	K. oxytoca	Human	R (>16)	R (>64/4)	R (>2)	R (>128)	S (≤1)	S (≤0.125)	S (≤4)	R (>2)

a R, resistant; I, intermediate; S, sensitive.

The presence of the CTX-M-15 ESBL gene in all human and environmental strains was confirmed by sequencing. The transfer of putative plasmids harboring these genes under laboratory conditions into wild-type E. coli strains via conjugation was not successful. In addition, no amplicons for *inc* genes (*incA/C*, *incL*, *incHI2*, *incM*, *incI1-αγ*, *incN*, *incFIIk*, *incFIA*, *incFIB*, and *incFII*) were obtained from the strains, indicating that the CTX-M genes might be chromosomally located, as described by Rodríguez et al. (20).

### Epidemiological links.

Retrospective analysis demonstrated that only newborns who had worn clothing that had been washed in the in-house washing machine were colonized with the redundant K. oxytoca clone. Although the siphons of the staff sinks were also identified as a possible reservoir, no staff members were identified as carriers or spreaders of ESBL-producing *Enterobacterales* at the time of screening.

All clinical and environmental isolates of PFGE type 00531/ST201 K. oxytoca displayed identical PFGE banding patterns and thus were considered clonally identical. This clone was specific for the newborns/infants and some environmental samples.

### Evaluation of possibly increased disinfectant tolerance of the K. oxytoca strains.

The environmental K. oxytoca isolates showed no increased tolerance to the surface disinfectant based on quaternary ammonium compounds and alkylamine. The alkylamine MICs for the K. oxytoca strains were identified as up to a maximum of 0.003%, compared to 0.0075% for the Pseudomonas aeruginosa reference strain. Furthermore, the examination according to Verbund für Angewandte Hygiene e.V. (VAH) method 9 showed reductions in the K. oxytoca strains of ≥5 log_10_ CFU at a concentration of 0.5% for 15 min in the presence of interfering substances (3.0 g/liter bovine serum albumin plus 3.0 ml/liter sheep erythrocytes [i.e., dirty conditions]). The VAH-recommended concentration and application time are 0.5% for 60 min (https://vah-online.de/en/for-users).

K. oxytoca isolates from the environment showed no increased tolerance to the laundry disinfectant based on peracetic acid. The MICs of the K. oxytoca strains and the P. aeruginosa reference strain were identified as 5 g/liter. The analysis according to VAH method 9 (in the presence of 2.75 g/liter laundry disinfectant with an exposure time of 15 min, under dirty conditions, at 20°C, 40°C, and 60°C) demonstrated that the K. oxytoca strains were more susceptible than the Gram-negative reference strain P. aeruginosa DSM 939. At a temperature of 60°C, all K. oxytoca strains showed reductions of ≥5 log_10_ units.

### Hygienic containment measures and control management strategies.

A task force was formed to manage the control interventions for prevention of the colonization of additional patients and to identify possible sources of the suspected K. oxytoca strain. The management procedures were divided into three main phases, until the colonizations were under control and the source could finally be identified. In each phase, step-by-step approaches with different strategies were necessary, in parallel with sampling during the whole reporting period (Fig. 1 and Table 3).

**TABLE 3:** Hygienic measures and control management procedures

Time period	Procedure(s)
April to August 2012	Environmental monitoring, including sampling of siphons, sinks, and showers via contact plates
	Continuation of admission screening and weekly routine screening and implementation of second newborn screening (48 h postpartum)
	Screening of all mothers at admission and at discharge
	Monitoring of pathways of newborns from birth to admission in the PNC
	Maintenance of incubator isolation until two-time negative screening results
	Advanced training of HCWs, with special focus on toilet and hand hygiene
	Microbiological analyses of vaginal ultrasound probes and heated towels used for primary care of newborns
	Renovation/decontamination of ward rooms and removal of unused sinks
	Installation of new wall-mounted disinfection dispensers
	Screening of clinical procedures for any cross-contamination possibilities
	Feeding of newborns with only precooked single-serving packages
	Use of sterile water for bathing of newborns
August to October 2012	Screening of all HCWs working in the PNC, ICU, or obstetrics department
October 2012 to June 2013	Extended environmental sampling from different areas in the ward and functionally linked areas in the hospital, with identification and sampling of specific risk factors (e.g., washing machine)
	Training of staff in correct handling of disinfectant wipes
	Training of HCWs in correct disinfection and cleaning of incubators
	Preparation of disinfectant wipes with newly validated/approved preparation machine
	Construction measures to provide work space for hygienic processing of incubators and to ensure sufficient storage capacities
	Marking of all thermometers (rectal and commercially available thermometers) and incubators for traceability
	Intensive audit of all procedures used in the ward during routine operations

### Long-term clinical effects and concomitant infection control interventions.

After the washing machine had been taken out of use, no further colonization of newborns with K. oxytoca has been detected to this day. All garments worn by newborns and children were laundered by a professional external hospital laundry service after the outbreak. The two colonized sinks were replaced by sinks with specialized thermosiphon systems. As a further consequence of the prolonged cluster process, the existing infection control measures (isolating colonized patients, enforcing hand hygiene measures, and cleaning the ward, particularly the sinks and equipment) were reinforced via extra training. The screening plan for all newborns and children and all measures implemented within the containment program continue to be maintained.

## DISCUSSION

### Reservoirs and transmission routes for waterborne pathogens in health care systems.

When the first 5 cases of colonization with K. oxytoca were detected, between August and October 2012, person-to-person transmission was suggested. Although this has not been reported for K. oxytoca, Price et al. (21) showed that, in the presence of standard infection control measures, HCWs were frequent sources of Staphylococcus aureus transmission. Furthermore, Heudorf et al. (22) mentioned that pathogens classified by the World Health Organization as highest priority can be found on staff gowns, which can be the starting point for transmissions (23).

In this case, the occurrence of K. oxytoca isolates continued over a 1-year period, which indicated a reservoir in the environment rather than among hospital personnel or mothers. Despite the strict implementation of control management and containment measures, newborns and children continued to acquire the cluster organism (PFGE type 00531/ST201), which had never been detected previously by the German National Reference Centre (GNRC). The single database strain was isolated in the Netherlands in 2018 (24).

### Washing machine.

In this case, the particular K. oxytoca strain was detected not only in two sinks but also in a domestic washing machine with an integrated dryer. Even recently published articles reviewing the main water-associated reservoirs in hospitals do not consider washing machines as potential hazards in clinical environments (4, 5). In our case, however, the knitted clothes of patients were washed in an in-house washing machine designed for household use and enabling a washing temperature of 65°C. It has been shown that resistance genes, as well as different microorganisms, can persist in domestic washing machines (17, 25).

Recently, a study suggested the potential role of washing machines in the distribution of antibiotic-resistant Gram-negative bacteria during laundering (17); until now, however, no transmission of pathogens from a washing machine to patients could be proven. In this case, we assume that the PFGE type 00531/ST201 K. oxytoca strain was disseminated to clothing after the washing process, via residual water on the rubber mantle and/or via the final rinsing process, which ran unheated and detergent-free water through the detergent compartment, although the water coming to the washing machine met the quality requirements of the hospital’s internal water controls. Consequently, we concluded that newborns were colonized by wearing hats and socks that had been contaminated by the washing process.

It remains unclear how the washing machine was contaminated. However, *Enterobacterales* species can survive in wet environments for extended periods (1), especially waterborne bacteria such as P. aeruginosa and *Klebsiella* strains, which have the ability to survive in a viable but nonculturable state. Their environmental stability is supported by the formation of biofilms to enhance their chances of multiplication and horizontal gene transfer (26). Zhang et al. (27) reported that biofilm detachment is promoted by disinfectants, thus affecting the overall antibiotic resistance of microbes in tap water.

Furthermore, Rehberg et al. (17) demonstrated that ARB can survive the washing process. In tests with P. aeruginosa outbreak strains, even the use of temperatures above 50°C could not achieve secure reduction of those strains. Although a 65°C washing program had been used for every wash cycle, it is likely that the temperature in the area of the rubber mantle or the rubber door seal would have been much lower, providing an optimal humid environment and nutrient supply for the growth of Gram-negative microorganisms (26, 28). Moreover, the occasional use of washing machines at low temperatures supports the formation of biofilms (14).

It is important to emphasize that the use of regular domestic washing machines for washing patients’ clothing in a clinical setting is not permitted, according to the current hygiene regulations in Germany. However, the washing machine concerned was located outside the actual central laundry preparation area and was intended only for washing mothers’ clothes and, as in this case, for washing the caps and socks; it was operated exclusively by the nursing staff.

### Conclusion.

While previous studies implicated sinks as potential reservoirs for clusters of infection caused by K. oxytoca, this report focuses on the washing machine. The extensive period of the outbreak shows that, with several potential environmental reservoirs, a multidimensional containment approach is necessary. Here, the approach included reinforcement of infection control policies, such as hand hygiene, contact precautions, isolation, admission/routine rectal screening, and clear delineation between handwashing sinks and sinks used for other purposes. However, the ongoing colonization was terminated only after the washing machine was removed and only disposable caps and socks were used. The results suggest that, for prompt management of outbreaks or colonization clusters, the choice of environmental sampling points and the use of effective methods should take into account the ecological properties of the causative strain. Indeed, the environmental strain was not found before the third on-site inspection, as the use of ESwabs allowed better detection.

In addition, the study implies that changes in washing machine design and processing are required to prevent the accumulation of residual water where microbial growth can occur and contaminate clothes. Furthermore, the use of professional washing machines and routine checking with a temperature logger are urgent requirements. In summary, the present study shows that, in situations where an increase in colonization is observed, nonprofessional washing machines, if used, and clothes should be assessed and investigated as potential reservoirs and vectors for transmission.

## MATERIALS AND METHODS

### Investigation strategy.

When the first colonizations were detected in April 2012, an initial management strategy to analyze the cases and to identify possible sources of the K. oxytoca strain was developed. Three major sampling events for all newborns, children, and mothers in the distinct wards took place during the reported time period, following a standard screening procedure.

In March 2013, sampling of specific risk areas (water/wastewater reservoirs) was performed, and ESBL screening (anal swabs and wound swabs) of all HCWs (physicians, nurses, and cleaning personal working in the PNC, the ICU, and the obstetrics department) was conducted to identify possible carriers. In June 2013, further sampling was conducted, using native samples (drinking water and wastewater) and liquid medium swabs (ESwabs).

### Laboratory analyses. (i) Microbiological cultivation.

Human and environmental samples were collected, stored at 4°C during transport to the laboratories, and processed within 48 h. All human samples (anal, vaginal, and wound swabs) were streaked onto MacConkey agar plates (Oxoid Deutschland GmbH, Wesel, Germany) with a 10-μg imipenem disk (bioMérieux SA, Marcy-Étoile, France) and selective agar plates, e.g., CHROMagar ESBL plates (Oxoid Deutschland GmbH). Oxoid Rodac contact plates (diameter of 55 mm; Becton, Dickinson, Heidelberg, Germany) were used for environmental surface sampling and were incubated at 36°C ± 1°C for 48 h. All environmental liquid medium swabs (ESwab; Copan Diagnostics, Italy) were streaked on Columbia agar plates containing 5% sheep blood (Becton, Dickinson), MacConkey agar plates (product no. PO5002A; Oxoid Deutschland GmbH), and selective agar plates (e.g., CHROMagar ESBL plates, product no. 43481; bioMérieux), and the plates were incubated at 37°C ± 1°C for 48 h. In addition, casein soy flour peptone broth (CASO broth) (Merck, Darmstadt, Germany) was inoculated with the same swabs and incubated at 37°C ± 1°C for 24 h.

The disinfectant wipes, socks, and knitted hats of the newborns were put into 200 ml of CASO broth with Tween-saponin-histidine-cysteine (TSHC) (3% Tween [product no. 8.22187; Merck], 3% saponin [product no. 5185.1; Roth], 0.1% histidine [product no. 1.04351; Merck], 0.1% cysteine [product no. 1.02839; Merck]) and homogenized for 60 s (Stomacher 400 blender; Seward Ltd., West Sussex, United Kingdom). Two 100-ml aliquots of the extract were filtered (Millipore Hydrosol membrane filter, with 250-ml cup), followed by the filtration of 100 ml of 0.9% NaCl to rinse the additives off the filter. The filter was then placed on CHROMagar ESBL and MacConkey agar plates, and the plates were incubated at 37°C for 24 to 48 h. In addition, 100 μl of each concentration of a dilution series in 0.9% NaCl was plated on CHROMagar ESBL and MacConkey agar plates. The emulsions were treated in the same manner as the wipes, except that shredding was not necessary.

For the water samples, approximately 100 ml was collected in a sterile polystyrene cup (product no. 225170; Greiner, Frickenhausen, Germany) and filtered through a sterile nitrocellulose membrane filter (pore size of 0.45 ± 0.02 μm and diameter of 47 mm, with a black grid, product no. EZHAWG 474; Millipore), according to the method described by Schulz and Hartung (29). After filtration, the membrane was placed on selective CHROMagar ESBL and MacConkey agar plates. In addition, 100 μl of each dilution of a dilution series in 0.9% NaCl was plated on CHROMagar ESBL and MacConkey agar plates.

### (ii) Identification and susceptibility testing of ARB.

All *Enterobacterales* strains detected on CHROMagar ESBL plates were identified by an API 20E system with Apiweb v4.1 (bioMérieux) and matrix-assisted laser desorption ionization–time of flight mass spectrometry (MALDI-TOF MS) (bioMérieux) using Myla software, the Vitek MS-α-cyano-4-hydroxycinnamic acid (CHCA) matrix (product no. 411 071; bioMérieux), and disposable targets (product no. 410 893; bioMérieux). Susceptibility to antibiotics was tested using the Micronaut-S MDR MRGN-Screening 3 system (MERLIN Diagnostika GmbH, Bornheim-Hersel, Germany), and results were interpreted using EUCAST criteria.

### (iii) Testing of disinfectant tolerance.

To detect possibly increased tolerance of the K. oxytoca isolates to disinfectants, surface disinfectant and the disinfecting general-purpose detergent that had been used in the washing machines were both examined according to the regulations (methods 7 and 9) of the Disinfectant Commission of the VAH (30). VAH method 7 was used to determine the MICs of the environmental K. oxytoca strains, in comparison with a Gram-negative reference strain, Pseudomonas aeruginosa DSM 939 (31). VAH method 9 is a quantitative suspension test that was used to identify efficacy gaps, taking into account the concentration-time relationship, interfering substances, and temperature of the disinfectants (32–34). The surface disinfectant used was based on quaternary ammonium compounds and alkylamine [didecyldimethylammoniumchloride, 12.5 g/100 g; bis-(aminopropyl)laurylamine, 1.5 g/100 g]. The laundry disinfectant was based on peracetic acid (40 to 80 ppm peracetic acid, 2.5 to 5 g/liter).

### Molecular resistance characterization. (i) Molecular characterization of ESBL genes.

Possible ESBL production was confirmed by PCR. Five specific primer sets were used to detect beta-lactamase-encoding genes belonging to the *bla*_TEM_, *bla*_SHV_, and *bla*_CTX-M_ families (35–38). The PCR products were visualized by gel electrophoresis on a 1% agarose-Tris-borate-EDTA (TBE) gel and stained with Midori green (Biozym Scientific GmbH, Hessisch Oldendorf, Germany). The resulting amplicons were purified using an innuPREP DOUBLEpure kit (Analytik Jena AG, Jena, Germany), according to the manufacturer’s recommendations. Custom sequencing was performed by Microsynth (Göttingen, Germany). The nucleotide sequences were analyzed using Chromas 2.6.5.

### (ii) Plasmid characterization (Inc typing).

To distinguish the Inc types of possible plasmids, 2 or 3 colonies of fresh cultures of all K. oxytoca isolates were resuspended in 100 μl of nuclease-free water, heated to 95°C for 10 min, and centrifuged at 14,000 × *g* for 5 min. The supernatant was carefully removed and used for PCR. The PCR mixture contained 2× OneTaq master mix (New England Biolabs) and 0.2 μM each primer (Table 4) (39–41).

**TABLE 4:** Primers used for Inc typing

Primer	Sequence	Reference
IncHI2_fw	TTT CTC CTG AGT CAC CTG TTA ACA C	Carattoli et al. (40)
IncHI2_rev	GGC TCA CTA CCG TTG TCA TCC T	Carattoli et al. (40)
IncA/C_fw	GAG AAC CAA AGA CAA AGA CCT GGA	Carattoli et al. (40)
IncA/C_rev	ACG ACA AAC CTG AAT TGC CTC CTT	Carattoli et al. (40)
IncL_fw	CGG AAC CGA CAT GTG CCT ACT	Carattoli et al. (41)
IncM_fw	GGA TGA AAA CTA TCA GCA TCT GAA G	Carattoli et al. (41)
IncL/M_rev	GAA CTC CGG CGA AAG ACC TTC	Carattoli et al. (41)
IncI1-αγ_fw	CGA AAG CCG GAC GGC AGA A	Carattoli et al. (40)
IncI1-αγ_rev	TCG TCG TTC CGC CAA GTT CGT	Carattoli et al. (40)
IncN_fw	GTC TAA CGA GCT TAC CGA AG	Carattoli et al. (40)
IncN_rev	GTT TCA ACT CTG CCA AGT TC	Carattoli et al. (40)
IncFIIk_fw	TCT TCT TCA ATC TTG GCG GA	Villa et al. (39)
IncFIIk_rev	GCT TAT GTT GCA CRG AAG GA	Villa et al. (39)
IncFIA_fw	CCA TGC TGG TTC TAG AGA AGG TG	Carattoli et al. (40)
IncFIA_rev	GTA TAT CCT TAC TGG CTT CCG CAG	Carattoli et al. (40)
IncFIB_fw	TCT GTT TAT TCT TTT ACT GTC CAC	Villa et al. (39)
IncFIB_rev	CTC CCG TCG CTT CAG GGC ATT	Villa et al. (39)
IncFII_fw	CTG ATC GTT TAA GGA ATT TT	Villa et al. (39)
IncFII_rev	CAC ACC ATC CTG CAC TTA	Villa et al. (39)

The PCR conditions were set at 94°C for 3 min, followed by 30 cycles of 94°C for 30 s, 60°C for 30 s, and 68°C for 1 min. The final extension was performed at 68°C for 5 min. The PCR products were analyzed using agarose gel electrophoresis on a 1% agarose-TBE gel and were stained with Midori green (Biozym Scientific GmbH).

### (iii) Plasmid conjugation testing.

The possibility of plasmid transfer via conjugation was analyzed. All K. oxytoca and wild-type E. coli strains were streaked onto LB agar containing (i) 20 mg/liter chloramphenicol or (ii) 20 mg/liter chloramphenicol and 4 mg/liter cefotaxime. E. coli strains that grew on LB agar containing only chloramphenicol but not on LB agar containing both chloramphenicol and cefotaxime were used for the following conjugation assay. For the preculture, 1 colony of fresh cultures of all isolated K. oxytoca strains (donors) and different wild-type E. coli strains (recipients) were used for inoculation of 5 ml lysogeny broth, and the broth was incubated overnight at 37°C, with shaking. Next, 5 ml of fresh lysogeny broth was inoculated with 100 μl of the donor (K. oxytoca) and 1 ml of the recipient (E. coli) and incubated overnight at 37°C, with shaking. After incubation, 1 ml of each culture was centrifuged at 14,000 rpm for 3 min. Subsequently, the supernatant was discarded and the pellet was resuspended using 100 μl of Milli-Q water. The whole suspensions, as well as 100 μl of the cultures, were plated onto selective LB agar containing 20 mg/liter chloramphenicol and 4 mg/liter cefotaxime and were incubated overnight at 37°C. Grown colonies were streaked onto Columbia sheep blood agar plates and analyzed via MALDI-TOF MS.

### (iv) Molecular typing.

All K. oxytoca strains isolated from clinical samples and environmental sources were typed by the GNRC using PFGE, according to the method described by Tenover et al. (42) and modified as described by Ribot et al. (43). In addition, multilocus sequence typing (MLST) of all K. oxytoca strains was conducted. Briefly, bacteria were cultivated overnight at 35°C on Columbia blood agar plates. After resuspension of 2 or 3 colonies in 80 μl of PCR water, the samples were heated to 95°C for 5 min and centrifuged at 14,000 × *g* for 5 min. The supernatant was used for PCR according to the protocol described by Herzog et al. (44). The PCR products were purified by extraction from a 1% agarose gel using the GeneJET gel extraction kit (Fisher Scientific GmbH, Schwerte, Germany), according to the manufacturer’s recommendations, and were sequenced by GATC Biotech AG (Constance, Germany). Assignment to the ST was performed via the specific MLST website (https://pubmlst.org/koxytoca).
